# Fisetin as a nutritional senotherapeutic: mechanisms, translational promise, and lessons from chronic kidney disease

**DOI:** 10.3389/fnut.2026.1867121

**Published:** 2026-09-21

**Authors:** Nur Warda B. Mohammed, David P. Baird, Eoin D. O'Sullivan

**Affiliations:** 1 Faculty of Health, Medicine and Behavioural Sciences, University of Queensland, Brisbane, QLD, Australia; 2 Population Health Program, QIMR Berghofer Medical Research Institute, Brisbane, QLD, Australia; 3 Centre for Inflammation Research, Institute for Regeneration and Repair, University of Edinburgh, Edinburgh BioQuarter, Edinburgh, United Kingdom; 4 Department of Renal Medicine, Royal Infirmary of Edinburgh, Edinburgh, United Kingdom; 5 Kidney Health Service, Royal Brisbane and Women's Hospital, Metro North, Brisbane, QLD, Australia

**Keywords:** aging, CKD - chronic kidney disease, fisetin, senescence, senolytic agent

## Abstract

Fisetin is a dietary flavonol that has attracted growing interest as a potential modulator of aging biology. In addition to its antioxidant and anti-inflammatory properties, experimental studies suggest that fisetin may influence cellular senescence through senomorphic and, in some contexts, senolytic actions. This has made it an attractive candidate within the wider field of food derived functional ingredients that target age related disease. Chronic kidney disease provides a useful case study for this emerging area because it is characterized by accelerated biological aging, chronic inflammation and fibrosis, and is associated with a high burden of frailty and multimorbidity. In this mini review, we summarize the nutritional and pharmacological features of fisetin, examine its proposed mechanisms of action, and discuss preclinical and early clinical evidence with a particular focus on kidney disease. We argue that fisetin is a promising but still unproven nutritional senotherapeutic. Its translation is limited by uncertainty around dietary exposure vs. pharmacological dosing, incomplete mechanistic understanding *in vivo*, bioavailability constraints, and sparse kidney specific clinical data. Chronic kidney disease highlights both the appeal and the difficulty of applying food derived bioactives to complex age-related disorders.

## Introduction

Cellular senescence has emerged as one of the central biological processes linking aging to chronic disease. Cellular senescence is a stable state of cell cycle arrest induced by cellular stress or aging, which is closely accompanied by pro-inflammatory secretion and metabolic dysfunction leading to altered tissue microenvironment and disease (1). This has driven growing interest in interventions that might modulate senescent cell burden or blunt its downstream effects. Among food derived bioactives, flavonoids have attracted particular attention because of their pleiotropic actions on oxidative stress, inflammation, mitochondrial function, and cell survival pathways. Fisetin, a dietary flavonol present in fruits and vegetables, has become especially prominent because experimental studies suggest it may act not only as an antioxidant and anti-inflammatory compound, but also as a senomorphic or senolytic agent in certain contexts. Chronic kidney disease (CKD) offers a useful case study for this emerging field. It is common, strongly associated with increasing age, and characterized by inflammation, fibrosis, frailty, and features of accelerated biological aging. CKD is a progressive condition characterized by irreversible loss of kidney structure and function, often driven by cumulative injury and maladaptive repair (1, 2). Here, we examine fisetin as a nutritional senotherapeutic, outline its biological rationale, and use CKD to illustrate both the promise and the current translational limits of flavonoid based approaches to healthy aging.

## Flavonoids

Flavonoids are a structurally diverse subclass of plant-derived polyphenolic secondary metabolites, ubiquitously present in fruits, vegetables, tea, and other plant-based foods. They are commonly subdivided into flavonols, flavanols, flavones, flavanones, anthocyanins, and isoflavones according to structural features. Fisetin is a dietary flavonol rather than a flavanol, and this distinction is important because different subclasses have different food sources, metabolism, and biological effects. Interest in fisetin has grown because it combines the broad redox and anti-inflammatory properties seen in many flavonoids with emerging evidence for more specific effects on senescence related pathways (4–12) (Table 1).

**Table 1:** Flavonoids share a common C6-C3-C6 diphenyl propane skeleton but are subdivided into distinct subclasses based on the degree of saturation and oxidation of the C-ring and the position of the B-ring attachment.

Subclass	Core structural feature	Representative compounds	Major dietary sources	Principal reported bioactivities
Flavonols	3-hydroxyflavone backbone 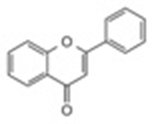	Fisetin, quercetin, kaempferol, myricetin	Onions, apples, strawberries, grapes, kale	Antioxidant, anti-inflammatory, emerging senolytic/senotherapeutic activity
Flavanols (flavan-3-ols)	Saturated C-ring, no 4-oxo group 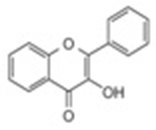	Catechin, epicatechin, epigallocatechin gallate (EGCG)	Tea, cocoa, apples, grapes	Endothelial function, vascular/antioxidant effects
Flavones	Unsaturated C-ring, 4-oxo group, no 3-OH 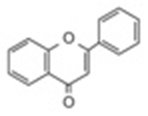	Apigenin, luteolin	Parsley, celery, citrus peel	Anti-inflammatory, antioxidant
Flavanones	Saturated C-ring with 4-oxo group 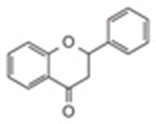	Naringenin, hesperetin	Citrus fruits	Anti-inflammatory, lipid-modulating
Anthocyanins	Flavylium (2-phenylchromenylium) cation 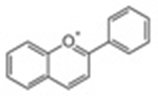	Cyanidin, delphinidin	Berries, red/purple grapes, red cabbage	Antioxidant, vascular protective
Isoflavones	Phenyl group at C3 rather than C2 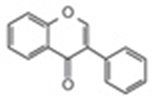	Genistein, daidzein	Soy, legumes	Phytoestrogenic, antioxidant

Fisetin is structurally distinct from flavan-3-ols (flavanols) despite the similarity in nomenclature, a distinction with functional relevance given the differing food sources, metabolic handling, and biological activities across subclasses. Structural diagrams depict the core scaffold characteristic of each subclass; substituent variation among the representative compounds listed (e.g., hydroxylation pattern, glycosylation) is not shown. Bioactivity data are drawn from in vitro, preclinical, and limited human studies as cited in the main text and are not necessarily subclass-wide or mechanistically equivalent across compounds.

Fisetin is one of several natural flavonoids proposed to have renoprotective or senotherapeutic activity. Quercetin, kaempferol as well as structurally unrelated plant-derived compounds have demonstrated antioxidant, and potential renoprotective effects (13–15). Quercetin is a flavonoid with structural similarity to that of fisetin, differing only by an additional 5-hydroxyl group on the A ring. Although structurally similar, previous work has demonstrated that fisetin and quercetin have different effects in inflammation and modulation of cancer cells (15–18). Relevant to kidney health, Quercetin appears to have a protective effect in animal models of kidney injury, mediated via reduced inflammation and downregulation of TGF-B signialling (19). As a senolytic however, Quercetin has only been studied in combination with Dasatinib, a tyrosine kinase inhibitor, which as brings the risk of side effects such as pulmonary arterial hypertension, pleural effusions and hematological toxicity (20, 21). A related compound, Kaempferol has shown promise *in vitro*, reducing oxidative stress and autophagy and some early mouse models of acute kidney injury (AKI), but human data are still lacking (22, 23).

Fisetin warrants particular consideration because it combines these pleiotropic effects with evidence of senolytic activity in selected cell types, without the requirement for coadministration of dasatinib for senolytic activity, and increasing renal exposure in murine pharmacokinetic studies and early human tolerability data (24). Fisetin has a higher anticancer potency than quercetin, demonstrates a greater senolytic activity, is commonly found in food and is available as an oral dietary supplement and thus has become the most promising flavonoid to explore therapeutically (3, 18).

Fisetin is hydrophobic molecule with an oral bioavailability of 44%, accumulating in cells to exert its effect potential effect *in vivo* (12, 25, 26). Mouse models have shown that it is rapidly distributed across tissues following oral dosing (27). It is rapidly bio transformed to fisetin glucuronides following oral administration. The rapid and terminal half-lives of fisetin are 0.09 h and 3.1 h respectively (28). In Murine tissue distribution studies, concentrations of fisetin and its active metabolites are highest in the kidneys but can be found distributed widely including in the brain within 2 h of administration (27, 28).

## How much fisetin do we consume in our diet

As a nutritional candidate, fisetin is attractive because it is naturally present in commonly consumed plant foods like strawberries, apples, grapes, onions, and cucumbers. Strawberries are observed to have the highest concentration of fisetin at ~150–160 μg/g whilst concentration in onions is more modest at ~4–5 μg/g. However, the nutritional relevance of fisetin requires careful interpretation. Habitual intake is likely to vary substantially according to dietary pattern, food source, ripeness, availability of seasonal plant foods (particularly strawberries, grapes and apples), and is almost certainly far lower than the doses used in most preclinical senescence studies (29). For example the STOP-sepsis clinical trial, administered fisetin at a dose of 20 mg/kg to prevent clinical deterioration in patients with Sepsis (30). This is at least ~3,500-fold higher than the typical average intake (31). This creates a persistent gap between fisetin as a component of an ordinary diet and fisetin as a putative therapeutic agent.

Food matrix effects are also likely to matter. As a hydrophobic flavonol, fisetin may differ in bioaccessibility according to formulation or molecular form, likely exhibiting variations in absortion in whole foods, extracts, or formulated supplements. Pure fisetin aglycone, the sugar-free, isolated form of the molecule, rather than from food-derived glycoside conjugatesas consumed in the diet, requires enzymatic cleavage in the gut prior to absorption, a step that is absent when pure aglycone is administered. Intestinal brush border enzymes, such as lactase-phlorizin hydrolase (LPH) and cytosolic β-glucosidase (CBG), hydrolyze flavonoid glycosides into their corresponding hydrophobic aglycones prior to cellular absorption. While fisetin is primarily consumed as a free aglycone rather than a glycoside, related dietary flavonoids rely heavily on this precise enzymatic deglycosylation step for transmembrane uptake Phase II metabolites like glucuronides and sulfates often possess lower intrinsic activity and rapid clearance compared to parent fisetin. Consequently, extensive conjugation drastically reduces systemic exposure to free fisetin, meaning *in vitro* efficacy cannot be directly extrapolated to *in vivo* tissues without accounting for active metabolite contributions or deconjugation at target site.

As a result Fisetin's low circulating parent-drug concentration does not necessarily represent its total biologically relevant exposure. Following oral administration in rodents, unchanged fisetin is present largely during the absorption phase, whereas sulfate and glucuronide conjugates subsequently predominate in serum (32). These conjugates retain measurable antioxidant activity, although they appear less potent than unconjugated fisetin (32). Fisetin is also methylated to geraldol, an active metabolite that has demonstrated cytotoxic and antiangiogenic effects and may achieve greater plasma exposure than the parent compound in mice (28, 33). Geraldol may possess senolytic, senomorphic or anti-fibrotic activity in animal models of lung disease but no data exists for such an effect in other organ systems including kidney cells or models (34). Consequently, as metabolites may well have relevant biological activity, low concentrations of unchanged fisetin cannot be equated directly with absent tissue activity, but the contribution of individual metabolites to its proposed renal effects remains uncertain. Future studies should quantify parent fisetin and its metabolites in plasma, urine and kidney tissue and directly compare their effects in relevant senescent renal cell populations.

This raises a key question for the field: are the observed anti-aging effects relevant to habitual nutrition, or do they mainly reflect pharmacological dosing of a food derived molecule? Framing fisetin solely as a dietary factor risks overstating the effect of ordinary intake, whilst conceptualizing it solely as a drug, neglects the nutritional context that makes it attractive in the first place. The data supporting dosing of Fisetin is summarized in Table 2.

**Table 2:** Fisetin dosing comparison - relative to Yousefzadeh et al. (3).

Study/ Source	Context	Form	Dose/ Concentration	Notes	Fold difference vs. Yousefzadeh et al. (3)^†^
Dietary exposure					
Kimira et al. (31)	Dietary	Food-derived glycoside	~0.4 mg/day (~0.006 mg/kg for 70 kg person)	Mean estimated intake from 3-day dietary survey; Japanese population	~16,700 × lower (0.006 vs. 100 mg/kg)
Food composition data ^*^	Dietary	Food-derived glycoside	~150–160 μg/g (strawberry) ~4–5 μg/g (onion)	Natural food-matrix concentrations	*Not directly comparable — food-matrix concentration, not systemic dose*
Preclinical — *in vitro*					
Zhu et al. (59)	*In vitro*	Pure aglycone	500 nM (apoptosis by caspase 3/7 in senescent HUVECs)	Senolytic activity cell-type specific: not observed in IMR90 fibroblasts or primary preadipocytes. In vitro/in vivo comparison approximate — see footnote^†^	~40 × lower (500 nM vs. 20 μM) [*in vitro* molar ratio only — see footnote^†^]
Yousefzadeh et al. (3)	*In vitro*	Pure aglycone	20 μM (human adipose explants & MEFs in culture)	Applied directly to tissue in culture medium	*IN VITRO REFERENCE (20 μM) In vitro/in vivo comparison approximate — see footnote^†^*
Preclinical — animal models					
Yousefzadeh et al. (3)	Mouse	Pure aglycone (diet)	500 ppm in chow (~50 mg/kg/day estimated)	Aged mice; intermittent — 2 weeks on, normal diet otherwise. 500 ppm estimated as ~50 mg/kg/day based on avg mouse chow intake (~3 g/day, ~30 g body weight)	~2 × lower (~50 vs. 100 mg/kg)
Yousefzadeh et al. (3)	Mouse	Pure aglycone (gavage)	100 mg/kg/day × 5 days	Aged mice (>20 months); decreased p16, p21, Cxcl2, Il10 in kidneys; extended healthspan	*IN VIVO* REFERENCE (100 mg/kg)
Ren et al. (80)	Mouse	Pure aglycone	50–100 mg/kg	Hyperuricaemic nephropathy model; near-normalization of BUN and creatinine	1–2 × lower to equal (50–100 vs. 100 mg/kg)
Safety data (refs (55–57))	Mouse	Pure aglycone	Up to 2,000 mg/kg LD50 oral: 1,700 mg/kg IP: 223 mg/kg/day × 14 days	No observable toxicity at 2,000 mg/kg; no mutagenicity on Ames test. Serum concentrations of 2.7–349.4 μM achieved *in vivo* without toxicity Zhu et al. (59)	Up to 20 × higher (2,000 vs. 100 mg/kg) [safety ceiling]
Clinical — human studies					
Hambright et al. (98)	Human	Pure aglycone (supplement)	100 mg/day (~1.4 mg/kg for 70 kg person)	Self-dosing; 35.6% decrease in senescent PBMCs; reduced SASP factors (MMP-3, MMP-9, IL-6)	~70 × lower (1.4 vs. 100 mg/kg)
Hodgin et al. (87); Lorenzo et al. (88)	Human	Pure aglycone (supplement)	500–1,000 mg/day (~7–14 mg/kg for 70 kg person)	Gulf War illness and prostate cancer trials; well tolerated	~7–14 × lower (7–14 vs. 100 mg/kg)
Krishnakumar et al. (86)	Human	Hybrid-hydrogel formulation	1,000 mg single dose (~14 mg/kg for 70 kg person)	15 healthy volunteers; enhanced bioavailability formulation; no significant adverse events	~7 × lower (14 vs. 100 mg/kg)
Silva et al. (30) (STOP-Sepsis; NCT05758246)	Human	Pure aglycone (oral)	20 mg/kg (~1,400 mg for 70 kg person) Single or two-dose course, 1 day apart	~3,500 × above mean dietary intake Kimura et al. (31); largest registered fisetin senolytic trial dose	5 × lower (20 vs. 100 mg/kg)

^†^All *in vivo* and clinical fold differences calculated relative to Yousefzadeh et al. (3) oral gavage dose of 100 mg/kg in aged mice. Human doses converted to mg/kg assuming 70 kg body weight. *in vitro* concentrations {Zhu et al. (59); Yousefzadeh et al. (3)} are in molar units and are not directly comparable to *in vivo* mg/kg doses; fold differences for *in vitro* rows reflect molar ratios between cell culture concentrations only.

## Lessons from chronic kidney disease

CKD is defined by persistent abnormalities of kidney structure or function for at least three months, including reduced estimated glomerular filtration rate (< 60 mL/min/1.73 m^2^), albuminuria, urinary sediment abnormalities, structural lesions, or histopathological evidence of kidney injury. CKD is classified according to cause, eGFR category, and albuminuria category, which together determine the risks of disease progression, cardiovascular complications, and kidney failure. CKD is a significant global health burden, affecting an estimated 10–13% of the world's population, resulting in millions of deaths worldwide and 1 in 57 fatal outcomes (35). With rising incidence attributed to an aging population, increasing rates of diabetes, and hypertension, the burden is set to increase in the future. Kidney disease is closely inter-related with heart and blood vessel disease, with 7% of all cardiovascular deaths attributed to CKD, and it remains an important cause of disability and disability-adjusted life years lost globally (36). It is projected to become the fifth leading cause of premature death worldwide by 2040 (37).

Clinically, CKD is characterized by progressive loss of filtration capacity and impaired tubular, endocrine, and homeostatic function, resulting in albuminuria, hypertension, fluid and electrolyte disturbances, metabolic acidosis, anemia, and CKD–mineral and bone disorder. Advanced disease may culminate in uraemic manifestations and kidney failure requiring dialysis or transplantation, while cardiovascular morbidity and mortality remain major competing risks throughout its course. Current management focuses on treating the underlying cause, controlling blood pressure and glycaemia, reducing albuminuria and cardiovascular risk. The mainstay of general treatment includees renin–angiotensin system blockade and sodium–glucose cotransporter-2 inhibitors while and managing disease-related complications. There is increasing roles for a multi modal disease modifying approach using medications such as finerenone and glucagon-like peptide-1 receptor agonists toprovide additional cardiorenal benefit in selected patients.

Beyond progressive loss of kidney function, the underlying pathophysiological changes in CKD mimic those seen with aging and CKD is increasingly recognized as a state of accelerated aging. Experimental and human observational data increasingly implicate cellular senescence in tubular injury, maladaptive repair, and fibrogenesis, making interventions that modulate senescence pathways a plausible target in CKD.

CKD provides a useful clinical context for evaluating fisetin because it sits at the intersection of aging biology, chronic inflammation, fibrosis, and frailty. It is not only a disease of declining organ function, but also one of accelerated biological aging, with systemic consequences that extend well beyond the kidney. Senescent cells accumulate in aged and injured kidneys, and growing evidence implicates senescence in tubular dysfunction, maladaptive repair, and fibrogenesis. As a result, CKD offers a clinically meaningful setting in which to test whether a food derived bioactive with putative senotherapeutic properties can move from mechanistic promise to translational relevance.

## The role of cellular senescence in CKD

Cellular senescence describes a state of irreversible proliferative arrest in cells with associated changes in chromatin organization, gene transcription, and protein secretion either *in vitro* or *in vivo* (38). Cellular senescence is accompanied by extensive chromatin remodeling, including the formation of senescence-associated heterochromatin foci, loss of lamin B1 (LMNB1), and redistribution of histone marks such as H3K9me3, which collectively alter transcriptional accessibility. These changes promote sustained expression of cell-cycle inhibitors, particularly CDKN2A and CDKN1A, while activating context-dependent inflammatory and profibrotic transcriptional programmes. A major functional consequence is the senescence-associated secretory phenotype (SASP), comprising cytokines and chemokines such as IL-6, IL-8/CXCL8, IL-1α, IL-1β, CCL2 and CXCL1; growth factors including TGF-β and VEGF; and extracellular matrix-remodeling enzymes such as MMP-3 and MMP-9. These mediators can reinforce senescence in an autocrine manner and propagate inflammation, fibrosis, immune-cell recruitment and tissue dysfunction through paracrine signaling.

Multiple studies have highlighted cellular senescence as a key contributor to age-related diseases, including CKD (39–41). The elderly and those with CKD have a high burden of senescent tubular epithelial cells and fibroblasts in the kidney (42). It is increasingly recognized that the burden of senescence correlates with worsening outcomes across a range of kidney diseases, including diabetic nephropathy, post AKI maladaptive repair leading to CKD, IgA nephropathy and post renal transplant (40, 43–46).

Fibrosis (i.e., scar formation) is characterized by excessive deposition of extracellular matrix leading to tissue scarring, representing the final common pathway of diverse chronic kidney diseases, in which fibrotic lesions arise not uniformly but at focal sites where a specialized fibrogenic niche recruits profibrotic signaling molecules such as Wingless-related integration site (WNTs) pathways and transforming growth factor-β to drive progressive tissue remodeling (47, 48). It is a fundamental component of CKD regardless of etiology and despite advances in recent years, there are no therapies that reverse or halt kidney fibrosis. Recent work has demonstrated that senescent cells are key drivers of kidney fibrosis (39, 43, 49, 50).

## Senolytic molecules target senescence

Senolytics are agents that selectively eliminate senescent cells from tissues and represent a promising therapeutic avenue for slowing CKD progression. Depletion of senescent cells improves organ function in animal models of aging and CKD suggesting that kidney senescent cells are potential therapeutic targets in human CKD (51–55). Early work used transgenic animals to deplete senescent cells and improve histological and function outcomes in mice (51, 52, 56). Pharmacological depletion of senescence cells has followed using a variety of promising agents to improve kidney histology and functional outcomes in animal studies (41, 53–55, 57). Senolytics are now being explored in multiple animal models and human phase 1 and 2 trials of dozens of varied, aged related diseases are underway (41, 58).

## Fisetin and dietary flavonoids as senolytic agents

### Mechanism of action

Fisetin exerts its senolytic effect via inhibition of BCL-2 pro-survival pathways in which anti-apoptotic proteins such as BCL-2, BCL-XL, and MCL-1 normally sequester the BH3 domains of pro-apoptotic effectors including BIM, BAK, and BAX to prevent activation of the intrinsic mitochondrial apoptosis pathway; by interfering with this binding, fisetin releases BAX/BAK-mediated mitochondrial outer membrane permeabilization, triggering the selective apoptotic clearance of senescent cells (59). *In vitro*, fisetin induces apoptosis of senescent human umbilical vein endothelial cells and senescent primary mouse embryonic fibroblast and reducing their numbers by 30–70% (3, 59). *In vivo* fisetin has reduced the number of senescent cells in adipose-derived stem cell without affecting the differentiation potential of remains cells (60). This degree of senolytic effect would be expected to have a measurable clinical effect based on genetic depletion in murine models, which alleviated an aged phenotype with 30% reduction in senescence cell number (61).

As a dietary flavonol, fisetin has antioxidant activity, acting as an activator of the Nrf2/antioxidant response element pathway, inducing downstream expression of HO-1 and elevating intracellular glutathione levels (62, 63). In aged mice, fisetin treatment restored hepatic glutathione ratios and reduced lipid peroxidation markers, demonstrating *in vivo* redox activity.4^2^ Fisetin also suppresses inflammatory signaling through inhibition of NF-κB and downstream SASP mediators including IL-6, IL-1β, and TNFα, with evidence of pathway modulation in both neuronal and peripheral tissues.7'55 This is further supported by preliminary human data; self-dosing of 100 mg/day fisetin was associated with reductions in circulating SASP factors including MMP-3, MMP-9, and IL-6, as well as a mean decrease of 35.6% in senescent peripheral blood mononuclear cells (60).

Fisetin's antioxidant, anti-inflammatory, anti-fibrotic and senotherapeutic activities are likely to represent interconnected rather than independent effects. Oxidative stress, mitochondrial dysfunction and lipid peroxidation can induce persistent DNA-damage signaling and tubular epithelial cell senescence. Senescent tubular cells then produce a SASP, including inflammatory and profibrotic mediators that activate NF-κB, recruit inflammatory cells and stimulate interstitial fibroblast proliferation, myofibroblast differentiation and extracellular matrix deposition (64–69). Fisetin-mediated activation of Nrf2 may reduce the oxidative stress that initiates or reinforces senescence, while inhibition of NF-κB may suppress the SASP without necessarily eliminating senescent cells. Suppression of SASP mediators, including IL-6 and TGF-β-associated signals, may attenuate fibroblast activation and TGF-β/SMAD3 driven fibrosis.

In relation to fibrotic disease, fisetin attenuates TGF-β/SMAD3 and IL-6/JAK2/STAT3 signaling in preclinical models of renal fibrosis (46, 49), reduces ferroptosis-associated tubular injury (47), and in a bleomycin-induced pulmonary fibrosis model, activated AMPK while inhibiting NF-κB and TGF-β/Smad3 signaling, reducing alveolar epithelial senescence, collagen deposition, and fibroblast-to-myofibroblast transdifferentiation (70). This suggests that fisetin's anti-fibrotic activity operates at least in part through senescence-dependent mechanisms, linking its senolytic and pleiotropic effects.

Ferroptosis is an iron-dependant form of regulated cell death driven by lipid peroxidation, and proximal renal tubule cells are particularly susceptible to this process, with ferroptosis increasingly implicated as a key contributor to tubular injury and disease progression in chronic kidney disease (71, 72). Ferroptosis forms an additional link between these pathways. Fisetin inhibits ACSL4-mediated tubular ferroptosis in experimental CKD, reducing lipid peroxidation, inflammation and fibrosis (73). Preventing ferroptotic tubular injury may reduce the generation of senescent cells and the subsequent SASP-driven fibrosis. However, the relationship is complex, as senescent tubular cells can become highly susceptible to ferroptosis, and experimental ferroptosis induction has selectively eliminated senescent tubular cells in some models (74). Conversely, ferroptotic stress can itself promote tubular senescence and age-related renal fibrosis (75). Thus, ferroptosis inhibition may be protective during ongoing tubular injury, whereas ferroptosis induction could theoretically eliminate an established senescent population. Overall, fisetin may interrupt the tubular injury–senescence–inflammation–fibrosis cycle at several points, but the relative importance and timing of this phenomenon remain unresolved.

The distinction between senolytic and senomorphic activity is also relevant to understanding fisetin's therapeutic profile. While senolytics remove senescent cells from a tissue, senomorphics affect their pathphysiological role without removing them, for example by suppressing supressing SASP expression such as by targeting IL- 1α, p38 MAPK and other signaling pathways (76). While fisetin acts primarily as a senolytic -selectively eliminating senescent cells via BCL-2/BCL-XL inhibition in an intermittent hit-and-run dosing paradigm (42, 43). It also demonstrates senomorphic SASP-suppressive activity, reducing secretion of IL-6 and IL-8 in senescent cells without inducing apoptosis (77). These two modes of action appears to be cell-type and context dependent, with senolytic efficacy demonstrated across Human Umbilical Vein Endothelial Cells (HUVECs), mouse embryonic fibroblasts, adipose-derived stem cells, and vascular endothelium, but not uniformly present across all cell types tested (42–44).

### Pre-clinical data

Evidence for kidney-specific activity of fisetin has emerged from murine models of kidney disease. Lupus nephritis is immune-mediated kidney inflammation caused by systemic lupus erythematosus (SLE), resulting from deposition of autoantibody-containing immune complexes within the glomeruli and other renal compartments. It typically presents with proteinuria, haematuria, active urinary sediment, and/or impaired kidney function (78). In lupus nephritis, fisetin reduced tubular epithelial cells expressing senescence-associated markers and was associated with fewer proliferating fibroblasts and α-smooth muscle actin-positive myofibroblasts, less collagen deposition and improved renal pathology (78). Similarly, in a cisplatin nephrotoxicity model, fisetin reduced persistent tubular senescence, increased apoptosis within chronically injured tubules, attenuated interstitial fibroblast activation and extracellular matrix accumulation, and improved tubular regeneration and renal function (79). Fisetin has also reduced fibrosis, SMAD3 phosphorylation, oxidative injury and inflammation in unilateral ureteric obstruction (29). Collectively, these studies strengthen the biological rationale for evaluating fisetin in CKD, particularly in disorders characterized by persistent tubular senescence and maladaptive repair and fibrosis.

Translating senescence modulation to kidney outcomes, Fisetin at doses of 50–100mg/kg resulted in an near normalization of kidney function (as measured by Blood urea nitrogen (BUN) and Creatinine) with no observable toxicity in a mouse model of hyperuricaemic nephropathy (80). In multiple other animal models,Cisplatin induced CKD, Unilateral ureteric obstruction(UUO) and Adenine diet-induced CKD, fisetin reduced ferroptosis, attenuated tubular injury, inflammation and fibrosis and senescence (73, 79). In aged mice (>20 months) fisetin decreased markers of senescence (including p16, P21, Cxcl2, Il10) in the kidneys and improved age related histological changes in murine kidneys (3). Fisetin has been shown to reduce fibrosis via inhibition of phosphorylation of SMAD3, oxidative damage, inflammation, apoptotic cell death, and accumulation of profibrotic M2 macrophages in injured, obstructed kidneys (29).

### Clinical data

As of January 2026, there were no new trials of fisetin (or any other flavonoid) underway in human CKD though there were 30 clinical trials underway testing the senolytic properties of flavonoids in other age-related diseases (81).

In an open-label phase 1 pilot study in 9 patients with diabetic kidney disease, a 3 day course of oral Dasatinib and Quercetin (a flavonoid similar to Fisetin) decreased senescent cell burden in adipose tissue, inflammation, fibrosis and circulating SASP factors for at least 11 days after the last dose of senolytics (82).

### Safety data

At doses up to 2000mg/kg fisetin has not shown any measurable toxicity signs in mice as well as no mutagenicity on an Ames test (83). Additionally, no toxicity symptoms were observed in the pathologic assessment of the animals heart, stomach, kidneys, intestine, liver, spleen, lungs and reproductive organs. A separate study using daily IP doses of 223 mg/kg daily for 2 weeks in mice, also demonstrated no toxicity (84). Oral LD50 values (i.e., the dose required to cause death in 50% of animals) of fisetin in mice were 1,700 mg/kg (oral) (85).

Fifteen healthy human volunteers taking 1,000 mg capsules reported no significant adverse events or clinical signs due to fisetin supplementation. Two cases of mild gastrointestinal side effects were reported - a “bloating sensation” and a decrease in appetite (86). Doses of 500–1,000 mg daily have been well tolerated in prior humans studies (87, 88). *In silico* analysis (pro-tox II tool, ADMET profiling using Toxcsm) supports the *in vivo* data, suggesting Fisetin is a molecule with minimal risk and low toxicity across a variety of parameters (89).

### Translational challenges and controversies

Despite growing interest in fisetin as a nutritional senotherapeutic, several translational challenges remain. First, there is an important tension between fisetin as a naturally occurring dietary compound and fisetin as a candidate therapeutic. Although fisetin is present in fruits and vegetables, the concentrations achieved through habitual dietary intake are likely to be substantially lower than those used in most experimental studies. This raises a fundamental question for the field: are the reported anti-aging effects relevant to normal nutritional exposure, or do they depend on pharmacological dosing of a food derived molecule. At present, the available evidence suggests the latter. No epidemiological studies have linked habitual dietary fisetin intake to any clinical or disease outcome, and the only population-level intake data derive from a Japanese dietary survey estimating mean daily fisetin consumption at approximately 0.4 mg/day, a figure that was not generated to assess health outcomes and falls orders of magnitude below the doses required to demonstrate senolytic or cytoprotective effects in experimental models (31). While broader cohort studies have associated higher total flavonol intake with reduced risk of dementia and cardiovascular disease, these findings cannot be attributed to fisetin specifically, given that fisetin constitutes only a minor fraction of total dietary flavonol intake even in produce-rich diets, and individual flavonols cannot be distinguished in population-level analyses (51). In the absence of fisetin-specific outcome data, it is not currently possible to attribute any clinical benefit to food-derived fisetin at nutritional concentrations.

Second, fisetin's mechanism of action *in vivo* remains incompletely defined. Experimental studies suggest that fisetin may reduce senescent cell burden, attenuate SASP signaling, and modulate oxidative, inflammatory, and fibrotic pathways. However, these actions may not be mutually exclusive, and the extent to which fisetin acts as a true senolytic, rather than as a broader cytoprotective or senomorphic compound, is still uncertain. This ambiguity is especially relevant in chronic disease states such as CKD, where inflammation, fibrosis, mitochondrial dysfunction, and metabolic disturbance overlap. It is plausible that fisetin exerts benefit through several parallel mechanisms, rather than through selective clearance of senescent cells alone.

Third, bioavailability and the relative roles of active metabolites remains uncertain. Fisetin is hydrophobic, undergoes rapid metabolism, and has a relatively short circulating half-life; these factors complicate extrapolation from preclinical models to human use. While tissue distribution studies suggest renal exposure may occur, it remains unclear whether sufficient concentrations can be achieved and sustained in relevant human tissues, particularly in people with kidney impairment. Future work should address formulation, bioavailable fisetin from various dietary sources, pharmacokinetics, metabolite activity, and the relationship between circulating exposure and tissue level biological effect.

CKD is associated with a highly variable gut microbiome environment, shaped by diet, transit time, medications, uraemia, constipation, antibiotics and comorbidity, and this can alter microbial metabolism, intestinal barrier function and production of uraemic solutes (90, 91). This is relevant to fisetin because orally administered flavonoids are partly dependent on intestinal handling and microbial enzymatic activity, meaning that hydrolysis, deconjugation, enterohepatic recycling and downstream metabolite generation may differ substantially between individuals. This is further complicated by the observation that fisetin itself modules the gut microbiome, which may be a source of therapeutic efficacy or variable bioavailability (92, 93). Fisetin may therefore modulate the gut–kidney axis, but these effects are likely to be unpredictable in CKD.

Formulation approaches may partially overcome issues with bioavailability of fisetin. Several delivery approaches have therefore been explored, including lipid-based systems, micelles, nanoparticles, liposomes, and hydrogel-based formulations (94–97). Liposome-encapsulated fisetin has been investigated as a strategy to improve cellular delivery and demonstrated senomorphic activity *in vitro*, including reduced inflammatory cytokine secretion in senescent-cell models. Hybrid-hydrogel formulations have also shown improved oral pharmacokinetic performance in healthy volunteers, supporting the concept that formulation can materially alter fisetin exposure (86).

Fourth, the clinical evidence base remains limited. Support for fisetin comes predominantly from *in vitro* work and animal models, with only early phase human studies and very limited kidney specific data. In CKD, this gap is even more pronounced. The biological rationale is strong, given the central roles of inflammation, fibrosis, frailty, and accelerated aging in kidney disease, but direct clinical data remains sparse. This makes CKD both an attractive and a demanding test case for translational research.

Finally, progress in this field will depend on better biomarkers and better trial design. Studies of senotherapeutics are hindered by the lack of universally accepted markers of senescent cell burden and target engagement in humans. In CKD, future studies may need to combine circulating and urinary biomarkers, tissue-based markers where feasible, frailty related endpoints, and conventional kidney outcomes to capture biological and clinical effect. Small mechanistic studies will likely be required before large scale efficacy trials can be justified. These should clarify whether fisetin is best studied as a dietary bioactive, a supplemental nutraceutical, or a pharmacological senotherapeutic

## Discussion

Fisetin sits at an interesting intersection between nutrition, aging biology, and translational medicine. As a food derived flavonol, it is appealing because it emerges from a broader nutritional framework rather than from conventional drug discovery alone. At the same time, the experimental literature suggests that fisetin may do more than simply reflect a healthy diet. Its reported effects on oxidative stress, inflammatory signaling, fibrosis, and cellular senescence have made it one of the most prominent candidate senotherapeutics within the flavonoid family.

CKD provides a particularly informative context in which to consider this potential. It is a disease of progressive organ dysfunction, but also one of accelerated biological aging, marked by frailty, inflammation, fibrosis, and high symptom burden. In this setting, fisetin is attractive not merely because it is a naturally occurring compound, but because its proposed biological actions map onto several of the core processes thought to drive kidney disease progression and its systemic consequences. Preclinical studies in kidney injury and aging models support this rationale, but the field is still developing. Evidence in humans is limited, kidney specific clinical studies are scarce, and the distinction between nutritional plausibility and therapeutic reality remains unresolved.

For these reasons, fisetin should be viewed as a promising but still unproven candidate whose value will depend on more rigorous translational work. Future studies should clarify the mechanisms most relevant *in vivo*, define pharmacokinetic and pharmacodynamic relationships in humans, and establish whether clinically meaningful effects can be achieved in populations characterized by accelerated aging, such as those with CKD. More broadly, fisetin illustrates both the promise and the difficulty of translating food derived bioactives into interventions that are mechanistically credible, clinically relevant, and realistic within nutrition science.
